# Tachycardia and persistent pulmonary hypertension of the newborn

**DOI:** 10.3389/fped.2025.1607907

**Published:** 2025-05-30

**Authors:** Ourania Kaltsogianni, Ravindra Bhat, Anne Greenough, Theodore Dassios

**Affiliations:** ^1^ Neonatal Intensive Care Centre, King’s College Hospital NHS Foundation Trust, London, United Kingdom; ^2^ Women and Children’s Health, School of Population and Life Course Sciences, Faculty of Life Sciences and Medicine, King’s College London, London, United Kingdom; ^3^ Neonatal Unit, University of Patras, Patras, Greece

**Keywords:** pulmonary hypertension, end-tidal carbon dioxide, arterial carbon dioxide, differential saturation, ventilation—mechanical

## Abstract

**Background:**

It is not known whether tachycardia in persistent pulmonary hypertension of the newborn (PPHN) is due to the use of inotropic agents or is a pathophysiological process related to the disease *per se*. In this study, we aim to test the hypothesis that tachycardia in PPHN would be related to non-invasive indices of PPHN.

**Methods:**

This is a retrospective study of ventilated infants with echocardiographically confirmed PPHN at the Neonatal Intensive Care Unit of King's College Hospital NHS Foundation Trust. The difference of the partial pressure of arterial (PaCO_2_) to end-tidal CO_2_ (EtCO_2_) or PaCO_2_–EtCO_2_ gradient was calculated as an index of PPHN severity and was related to the level of tachycardia at acute PPHN and after the resolution of PPHN (pre-extubation).

**Results:**

Fifteen infants with PPHN were studied, whose median (interquartile range, IQR) gestational age was 35.7 (34.1–40.3) weeks and birth weight was 2.95 (2.17–3.20) kg. It was found that the median (IQR) heart rate was higher during acute PPHN [158 (122–169) bpm] compared with that during pre-extubation [119 (111–136) bpm, *p* = 0.016]. The difference in the heart rate at acute illness and pre-extubation was significantly related to the difference in the PaCO_2_–EtCO_2_ gradient (correlation coefficient = 0.732, *p* = 0.016).

**Conclusions:**

There was a significant association between tachycardia and the severity of PPHN in ventilated infants, possibly describing that tachycardia is the result of PPHN *per se*, as well as the effect of inotropes.

## Introduction

Persistent pulmonary hypertension of the newborn (PPHN) is characterised by a failure of the pressure in pulmonary circulation to decrease to a level below systemic blood pressure after birth, causing significant right-to-left shunting via the foramen ovale and/or the ductus arteriosus and subsequent refractory hypoxaemia (1). The treatment goals of PPHN include lowering the pulmonary pressure with targeted interventions such as the use of inhaled nitric oxide and optimisation of the systemic blood pressure with the use of inotropic agents (2). The choice of inotropes is influenced by the cardiovascular state; for example, a high heart rate would preclude the use of chronotropic agents such as dobutamine or adrenaline, as they would probably increase the levels tachycardia (3).

Other than inotropes, however, tachycardia in PPHN might also be the result of the disease, as high pulmonary pressure and the ensuing increased right-to-left shunting would cause a hyperdynamic circulation and subsequent tachycardia. Determining whether tachycardia in PPHN is secondary to inotropes or the disease *per se* is important, as it could influence the decision to use inotropes and affect treatment efficiency. PPHN is often assessed by differential saturation or differential cyanosis (the difference between pre-ductal and post-ductal transcutaneous oxygen saturations, signifying ductal right-to-left shunting). We have also described another non-invasive index of disease severity in PPHN: the difference of the partial pressure of arterial (PaCO_2_) to end-tidal CO_2_ (EtCO_2_) or PaCO_2_–EtCO_2_ gradient (4). This gradient is increased secondary to the recirculation of CO_2_ during PPHN with right-to-left shunting. A strong correlation of the level of tachycardia with those non-invasive indices of PPHN might point towards the conclusion that tachycardia can be the result of the disease *per se*.

We hypothesised that the PaCO_2_–EtCO_2_ gradient and the level of differential cyanosis would be significantly related to the level of tachycardia in PPHN. Our aim was to test this hypothesis.

## Materials and methods

A retrospective, observational study of ventilated infants [greater than 32 weeks of gestational age (GA)] with echocardiographically confirmed PPHN with right-to-left shunting at the Neonatal Intensive Care Unit of King's College Hospital NHS Foundation Trust (KCH) was undertaken. Infants were recruited between October 2023 and March 2025. PPHN was diagnosed based on echocardiographic parameters for the assessment of pulmonary artery pressure, pulmonary vascular resistance, right ventricular performance, and shunt (5). The infants with PPHN underwent echocardiography for clinical confirmation of PPHN, which was suspected because of >5% difference between pre- and post-ductal transcutaneous saturation and/or an oxygen requirement of more than 50%, which was not considered to be in accordance with the radiographic severity of the lung disease. The infants were ventilated with the SLE 6000 Neonatal Ventilator (Inspiration Healthcare, Crawley, UK) on volume targeted ventilation with a targeted volume of 5–6 ml/kg (6). Inhaled nitric oxide was started at 20 ppm and weaned according to clinical response. Ventilated infants with PPHN were given intravenous sedation with morphine at infusion rates of 5–20 μg/kg/h. The study was registered with the clinical governance department at KCH as part of a service evaluation of the use of nitric oxide in ventilated infants. The Health Research Authority Toolkit of the National Health System, United Kingdom, confirmed that the study was not considered research and hence would not need regulatory approval by a research ethics committee.

### Assessment of the PaCO_2_–EtCO_2_ gradient

The PaCO_2_–EtCO_2_ gradient (in mmHg) was calculated concurrently with the echocardiographic assessment, as there existed a difference between the partial pressure of arterial carbon dioxide measured from indwelling arterial sample lines and end-tidal carbon dioxide measured by continuous Microstream sidestream capnography (6). The PaCO_2_–EtCO_2_ gradient was recorded during the acute illness (when the echocardiography was performed) and when PPHN had clinically resolved. This was on the day the infants were deemed by the clinical team to be ready for extubation, when they had an oxygen requirement of less than 40%, a pH >7.25 and a PaCO_2_ <8.5 kPa, and their breathing rate was above the set ventilator rate.

### Clinical information

The following information was collected from the medical notes: GA (weeks), birth weight (BW, kg), sex, Apgar score at 5 min, mode of delivery (vaginal or caesarean section), diagnosis related to PPHN, and the use of inhaled nitric oxide (yes/no). The following information was collected at the two time endpoints (acute illness with PPHN and pre-extubation) by averaging the hourly recorded values 4 h around the echocardiogram, which diagnosed PPHN, or 4 h before extubation: heart rate, pre-ductal transcutaneous oxygen saturation (SpO_2_), post-ductal SpO_2_, and systemic blood pressure (mmHg). The differential saturation was calculated as the difference in the pre-ductal minus the post-ductal SpO_2_. The oxygenation index was also calculated from the corresponding values of the fraction of inspired oxygen, mean airway pressure (in cmH_2_O), and arterial partial pressure of oxygen (in mmHg) (7). The following inotropes were administered as continuous infusions as per the unit guideline: noradrenaline (20 ng–1 μg/kg/min), adrenaline (50 ng/kg/min–1 μg/kg/min), milrinone (0.25–0.75 μg/kg/min), dobutamine (2–20 μg/kg/min), and dopamine (2–20 μg/kg/min).

### Statistical analysis

As PPHN is an uncommon condition and there is a paucity of published data on the PaCO_2_–EtCO_2_ gradient in infants with PPHN, we aimed to recruit a convenience sample of all infants with echocardiographically confirmed PPHN admitted to the neonatal intensive care unit (NICU) during a recent 18-month period. Data were tested for normality and found to be non-normally distributed. Data were thus presented as median and interquartile range (IQR) and non-parametric tests were utilised to determine whether differences were statistically significant (*p* < 0.05). The heart rate, differential saturation, and PaCO_2_–EtCO_2_ gradient were compared at PPHN and before extubation using the Wilcoxon rank sum test for related samples. The difference in the heart rate at acute illness and before extubation (delta HR) was calculated as the HR at acute illness minus the HR pre-extubation. Similarly, the difference in the differential saturation (delta differential SpO_2_) and the difference in the PaCO_2_–EtCO_2_ gradient (delta PaCO_2_–EtCO_2_ gradient) were calculated by subtracting the differential saturation pre-extubation from the differential saturation at acute illness and the PaCO_2_–EtCO_2_ gradient pre-extubation from the PaCO_2_–EtCO_2_ gradient at acute illness, respectively. To examine the relationship of the change in HR with the changes in the differential SpO_2_ and the PaCO_2_–EtCO_2_ gradient, the delta HR was correlated to the delta differential SpO_2_ and the delta PaCO_2_–EtCO_2_ gradient using Spearman's Rho correlation analysis.

Statistical analysis was undertaken with SPSS version 28.0 (SPSS Inc., Chicago, IL, USA).

## Results

Fifteen infants (five male) were studied with a median (IQR) gestational age of 35.7 (34.1–40.3) weeks and a birth weight of 2.95 (2.17–3.20) kg. The diagnoses related to PPHN were that four infants had meconium aspiration syndrome, four had congenital diaphragmatic hernia, two had hypoxic ischaemic encephalopathy, one exomphalos, one had congenital intestinal atresia, one sepsis, one had Trisomy 21, and one was idiopathic without proven concomitant pathology. Ten infants (67%) were born via emergency Caesarean section. The infants had a median (IQR) Apgar score of 9 (6–10). Fourteen infants were treated with inotropes at the point of echocardiographical diagnosis of PPHN. The specific inotropes used included noradrenaline (12 infants), adrenaline (9 infants), milrinone (7 infants), dobutamine (4 infants), and dopamine (2 infants). All infants were treated with inhaled nitric oxide during acute illness and were weaned off nitric oxide by the point of extubation.

**Figure 1:**
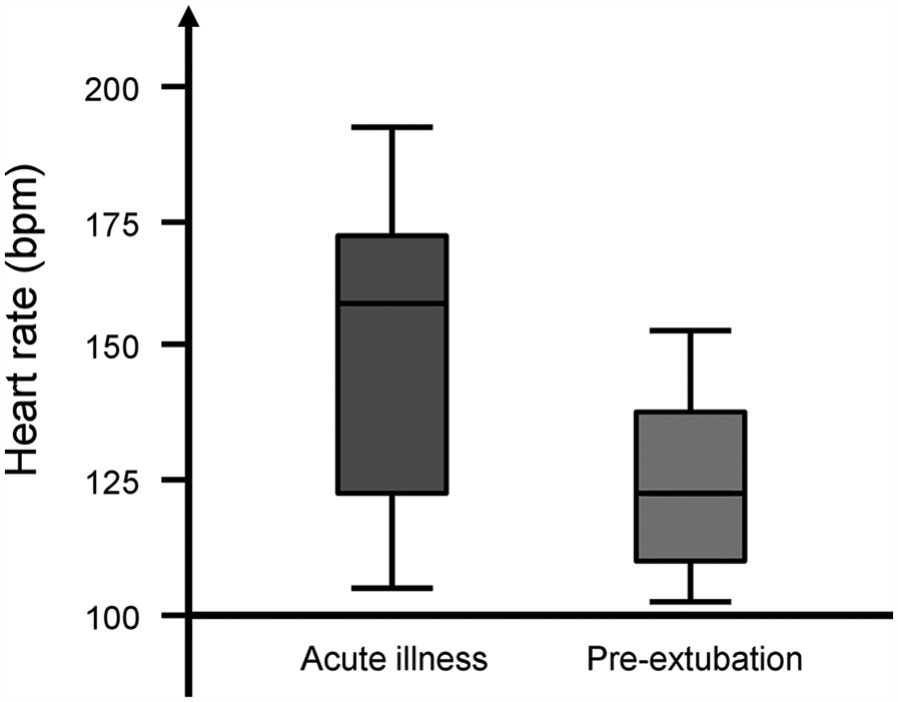
Box plot of the heart rate of infants with persistent pulmonary hypertension at acute illness and pre-extubation. The horizontal lines represent the minimum, lower quartile, median, upper quartile, and maximum values.

The median (IQR) heart rate was higher during the acute illness [158 (122–169) bpm] compared with that during pre-extubation [119 (111–136) bpm, *p* = 0.016, Figure 1]. The median (IQR) differential saturation was higher during the acute illness [1 (0–2)] compared with that during pre-extubation [0 (0–0), *p* = 0.043]. The median (IQR) PaCO_2_–EtCO_2_ gradient was not significantly different during the acute illness [10 (7–12)] compared with that during pre-extubation [8 (4–11), *p* = 0.507, Table 1].

**Table 1:** Cardiorespiratory parameters at acute illness and pre-extubation.

	Acute illness	Pre-extubation	*p*-value
Age (days)	1 (1–18)	11 (7–27)	0.016
Heart rate (bpm)	158 (122–169)	119 (111–136)	0.016
PaCO_2_ (mmHg)	44 (39–49)	42 (32–46)	0.953
EtCO_2_ (mmHg)	36 (30–39)	34 (30–38)	0.374
PaCO_2_–EtCO_2_ gradient (mmHg)	10 (7–12)	8 (4–11)	0.507
Pre-ductal SpO_2_ (%)	97 (93–98)	95 (94–98)	0.593
Post-ductal SpO_2_ (%)	96 (94–98)	97 (96–98)	0.173
Differential saturation (%)	1 (0–2)	0 (0–0)	0.043
Systemic blood pressure (mmHg)	50 (47–56)	59 (55–62)	0.009
Oxygenation index	13.2 (6.1–20.6)	4.3 (2.3–6.6)	0.043

PaCO_2_, arterial partial pressure of carbon dioxide; EtCO_2_, end-tidal carbon dioxide; SpO_2_, transcutaneous oxygen saturation.

Data are presented as median (IQR).

The median (IQR) delta HR was 14 (7–31) bpm (range: 0–58 bpm), the delta differential SpO_2_ was 2% (1%–3%), and the delta PaCO_2_–EtCO_2_ gradient was 3 (0–9) mmHg. The delta HR was significantly related to the delta PaCO_2_–EtCO_2_ gradient (rho = 0.732, *p* = 0.016) but not to the delta differential SpO_2_ (rho = 0.378, *p* = 0.530).

## Discussion

We have demonstrated that infants with PPHN presented with a significantly higher heart rate at acute illness compared with that at pre-extubation and that the decrease in their heart rate was associated with non-invasive indices of PPHN such as the gradient of the arterial to end-tidal carbon dioxide. This relationship possibly highlights a pathophysiological link between the two phenomena and that tachycardia is a manifestation of the disease rather than only a transient side effect of inotropic agents.

Although tachycardia is often included in the literature as a sign of PPHN, prior to our study, there was little published evidence to substantiate this claim (8). Interestingly, in our cohort, the difference in the heart rate was significantly associated with the carbon dioxide gradient, but this relationship was not found between the heart rate and the differential saturation. It is plausible that the differential saturation is a less sensitive marker of PPHN compared with the carbon dioxide gradient, as it can only detect differences that are due to shunting at the ductal level and cannot detect shunting at the level of the foramen ovale (4). Contrarily, the difference in the carbon dioxide gradient would detect any recirculation of carbon dioxide due to blood mixing at both the ductal and the atrial levels (4). It was also notable that, in our study, the carbon dioxide gradient did not significantly decrease pre-extubation compared with the value measured at acute illness and that the median value dropped only from 10 to 8 mmHg. This is not in agreement with what we have reported in the past in another cohort: that the resolution of PPHN was associated with a significant decrease in the CO_2_ gradient and a lowering of the median values from 10.7 to 3.7 mmHg (4). These differences might be explained by population differences, as in our previous study, we had included infants only with idiopathic PPHN, while in this study, we included infants with secondary pulmonary hypertension, which would be expected to be due to a fixed and structural element of pulmonary vascular disease (such as due to meconium aspiration syndrome, congenital diaphragmatic hernia, exomphalos, or trisomy 21) (9). In those cases, the resolution of PPHN might not occur in a short period of time, such as the one we included in our study, and corresponds to the relatively brief period of invasive ventilation.

In this study, we report only a pathophysiological association between PPHN and tachycardia, and we cannot prove unequivocally that PPHN *per se* is the cause of tachycardia. It is possible that the employed inotropic agents exerted an independent action on the heart rate, irrespective of the disease progression (10). The methodological approach that would address this uncertainty would be to randomise infants with PPHN to either receiving inotropes or not, but this process would be clinically questionable, as these infants are usually acutely severely unwell, and withholding a potential treatment would not be welcomed by most clinicians. Based on our results, we cannot suggest that tachycardia should not be considered when the choice of inotropes is made, but we highlight that this phenomenon might be an intrinsic structural component of the disease and might persist as a sign of the severity of the disease irrespective of the choice of inotropes or even their use at all.

The strength of our study is that it was a pragmatic attempt to approach a real clinical problem. We acknowledge as a limitation that we report a small single-unit retrospective sample with a heterogeneous population consisting of infants with different primary aetiologies for their PPHN. This is, however, the clinical context in which PPHN presents (11), and the rarity of the complication would make it unfeasible to recruit a much larger sample in a predefined recent chronological period. Future studies could investigate the relationship between nitric oxide administration and the level of tachycardia.

With regard to the relationship of tachycardia with specific inotropic agents, it is important to remember the force–frequency relationship as an intrinsic regulatory mechanism of cardiac contractility. This mechanism describes that an increase in contractile force is induced by an elevation of the stimulation frequency (12). Finally, centres that use targeted neonatal echocardiography and consider physiological principles tend to choose vasopressors with a favourable pulmonary vascular profile and avoid extreme or prolonged tachycardia (13).

In conclusion, we have described a significant association between a high heart rate and the severity of PPHN in ventilated infants, possibly describing that tachycardia is the result of PPHN *per se*, as well as of the use of inotropes in this population.

## Data Availability

The data analyzed in this study is subject to the following licenses/restrictions: Data will be shared upon reasonable request. Requests to access these datasets should be directed to theodore.dassios@kcl.ac.uk.
